# FgMsn2, a zinc finger transcription factor, regulates stress responses, pathogenicity and metabolism in wheat scab fungus *Fusarium graminearum*

**DOI:** 10.1007/s44154-025-00249-2

**Published:** 2025-09-02

**Authors:** Daiyuan Sun, Chengliang Li, Liangyuan Zhao, Jinling Yang, Haijuan Li, Kaili Duan, Chenfang Wang, Guanghui Wang

**Affiliations:** 1 State Key Laboratory for Crop Stress Resistance and High-Efficiency Production, College of Plant Protection, Northwest A&F University, Yangling, 712100 Shaanxi China; 2 Institute of Plant Protection, Beijing Academy of Agriculture and Forestry Sciences, Beijing, 100097 China; 3 College of Biological and Environmental Engineering, Xi’an University, Xi’an, 710065 Shaanxi China

**Keywords:** Deoxynivalenol (DON), Transcription factor, Stress Responses, Mitochondrial morphology, Lipid metabolism, *Fusarium graminearum*

## Abstract

Environmental stress adaptation is crucial for the survival and pathogenicity of plant fungal pathogens. In this study, we identified a transcription factor FgMsn2 in *Fusarium graminearum*, an ortholog of Msn2 in budding yeast. Structural analysis showed that the C2H2 zinc-finger domain is highly conserved across fungi, while other regions are less conserved, suggesting that FgMsn2 may have species-specific functions. Subsequently, we revealed that FgMsn2 is critical for vegetative growth, and conidiogenesis. Deletion of *FgMSN2* severely reduced the deoxynivalenol (DON) production and pathogenicity, while enhancing tolerance to oxidative, osmotic, cell wall and membrane stresses. Furthermore, our RNA-seq analysis revealed that FgMsn2 regulates genes involved in energy metabolism, lipid metabolism and stress responses, emphasizing its role in maintaining metabolic balance and stress adaptability. Notably, FgMsn2 influences mitochondrial morphology, as the *Fgmsn2* mutant exhibited disrupted mitochondrial structures and reduced ATP production. The *Fgmsn2* mutant also showed increased lipid droplet accumulation, indicating the FgMsn2’s role in lipid metabolism. Taken together, the FgMsn2 serves as a key regulator in fungal development, plant infection, stress responses, and metabolism. Our study provides valuable insights into the molecular mechanisms of fungal stress adaptation and pathogenicity, suggesting a potential target for the development of more effective fungicides and disease management strategies.

**Supplementary Information:**

The online version contains supplementary material available at 10.1007/s44154-025-00249-2.

## Introduction

*Fusarium graminearum* is the major pathogenic fungus that infects wheat, causing *Fusarium* head blight (FHB) (Goswami and Kistler 2004). This devastating disease significantly reduces crop yield and quality, leading to severe economic losses in agricultural production (Mcmullen et al. 1997). Moreover, *F. graminearum* contaminates grains with harmful mycotoxins, such as deoxynivalenol (DON) and zearalenone (ZEN), posing serious health risks to humans and animals (Brown et al. 2004). DON toxin is also an essential virulence factor, promoting *F. graminearum* to cross the rachis node during wheat head infection (Proctor et al. 1995). The fungus depends on both DON toxin production and its rapid response to environmental or host-induced stresses for its survival and pathogenicity under various conditions.

In nature, pathogenic fungi encounter various challenges, such as heat shock, oxidative stress, osmotic stress, and host-induced stress, which they must overcome to survive and maintain their pathogenicity (Zhang et al. 2021). To adapt, they have developed sophisticated stress response mechanisms, including stress signal recognition, transduction, and precise transcriptional regulation (Gasch 2007; Zhang et al. 2021; Lin et al. 2022). The C2H2-type zinc finger transcription factor Msn2 was first identified in *Saccharomyces cerevisiae* (Martinez‐Pastor et al. 1996) and has been extensively studied in yeast and filamentous fungi. Notably, the Msn2 is a core component of the fungal stress response network, enabling fungi to cope with environmental challenges (Martinez‐Pastor et al. 1996; Liu et al. 2013; Zhang et al. 2014; Freitas et al. 2016). In *S*. *cerevisiae*, Msn2 along with its paralog Msn4, binds to stress response elements (STREs) to regulate the stress-related genes under stress conditions (Martinez‐Pastor et al. 1996). While single mutants of *msn2* or *msn4* show minimal phenotypic changes, double mutants are highly sensitive to various stresses (Martinez‐Pastor et al. 1996), indicating their overlapping and redundant roles in stress responses.

In some fungi, the Msn2 plays a crucial role in responding to oxidative, osmotic, cell wall, and heat shock stresses. However, in *Candida albicans* and *Verticillium dahliae*, Msn2 appears to play no role in stress responses (Nicholls et al. 2004; Tian et al. 2017), suggesting that they may utilize alternative mechanisms for stress adaptation. Interestingly, in *Candida glabrata*, a close relative of *C. albicans*, the Msn2/4 play an important role in stress responses (Roetzer et al. 2008), highlighting a species-specific functional divergence within the same genus. Moreover, in some fungi, the deletion of *MSN2* unexpectedly endows them with enhanced stress tolerance. For instance, the *Momsn2* deletion mutant in *Magnaporthe oryzae* exhibits increased resistance to cell wall stress, induced by Calcofluor White (Zhang et al. 2014). Similarly, in *Alternaria alternata*, deletion of *AaSRR1*, the *MSN2* ortholog, enhances resistance to multiple oxidative stresses as well as cell wall and membrane stresses (Lu et al. 2022). Therefore, Msn2 orthologs exhibit significantly varied stress response functions in different fungal species.

The Msn2 orthologs are also involved in pathogenesis and development in pathogenic fungi. Notably, Msn2 is indispensable for pathogenicity in many fungal pathogens, including *M. oryzae, Valsa mali*, *Botrytis cinerea*, and *Beauveria bassiana* (Liu et al. 2013; Zhang et al. 2014; Wu et al. 2018; Lu et al. 2023). Furthermore, Msn2 orthologs regulate conidiation in various fungi, although their regulatory roles differ depending on the species. For instance, in *M*. *oryzae* and *Ustilaginoidea virens* they act as positive regulators (Zhang et al. 2014; Xu et al. 2021), whereas in *A. alternata* and *Aspergillus flavus* they function as negative regulators (Chang et al. 2011; Lu et al. 2022). Moreover, in the entomopathogenic fungus *Metarhizium rileyi*, the MrMsn2 negatively regulates the yeast-to-hypha transition, a process critical for infecting host and evading immune responses (Song et al. 2018). In contrast, in the oleaginous yeast *Yarrowia lipolytica*, the Mhy1, an Msn2/4-like protein, positively regulates dimorphic switching (Hurtado and Rachubinski 1999). In addition, Msn2 regulates secondary metabolism. Fungi rely on these secondary metabolites for ecological adaptation, environmental competition, and interactions with other organisms (Macheleidt et al. 2016). In *Aspergillus parasiticus* and *A*. *flavus*, the *msnA* mutants produce increased levels of aflatoxins and kojic acids, which are believed to help mitigate the increased oxidative stress within the cells (Chang et al. 2011). Similarly, in *U. virens*, UvMsn2 regulates the biosynthesis of phytotoxic compounds, thereby enhancing pathogenicity (Xu et al. 2021).

The function of Msn2 ortholog (FgMsn2) in *F. graminearum* remains unknown, despite its characterization in some yeast and filamentous fungi. In this study, we generated the *Fgmsn2* deletion mutant using a gene knockout approach, and revealed that FgMsn2 regulates multiple biological processes, such as hyphal growth, conidiation, pathogenicity, and DON synthesis. Moreover, FgMsn2 plays an important role in response to various environmental stresses, including osmotic, oxidative, and cell wall and membrane stresses. Importantly, we also identified the pivotal role of FgMsn2 in orchestrating mitochondrial dynamics and lipid metabolism, suggesting its function as a key regulator in metabolic processes.

## Results

### Identification of Msn2 in *F. graminearum*

**Fig. 1:**
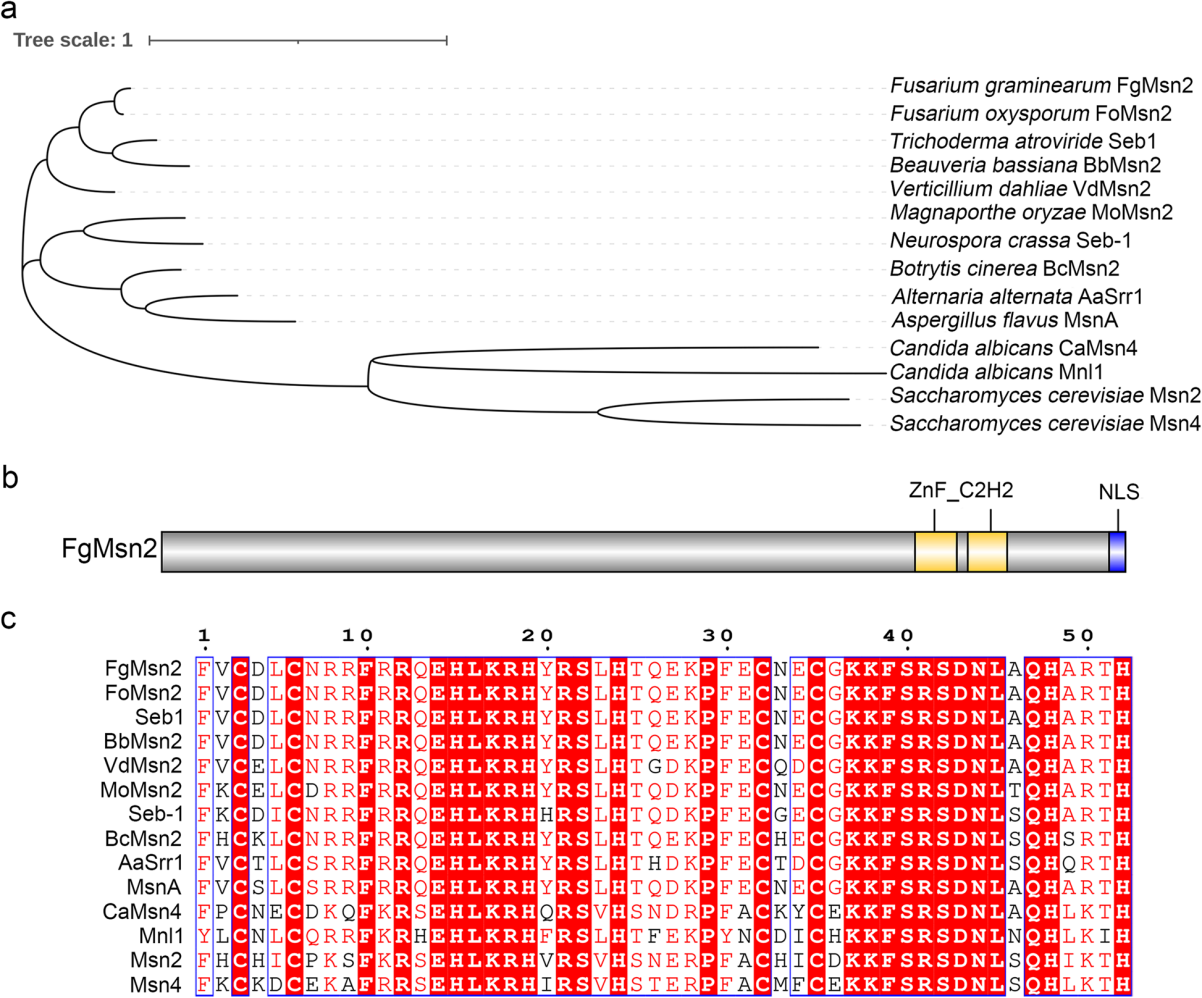
Structure and phylogenetic analysis of FgMsn2 **a** Schematic drawing of phylogenetic analysis with Msn2 orthologs from *F. graminearum* (FGSG_06871), *F. oxysporum* (FOXG_01955), *T. atroviride* (TRIATDRAFT_135877)*, B. bassiana* (BB8028_0003g08640), *V. dahlia* (VDAG_04227), *M. oryzae* (MGG_00501), *N. crassa* (NCU02671), *B. cinerea* (Bcin01g05160), *A. alternata* (AA0117_g7840), *A. flavus* (COH20_001118), *C. albicans* (C1_08940C_A, CaMsn4), (CR_07450C_A, Mnl1) and *S. cerevisiae* (YMR037C, Msn2), (YKL062W, Msn4). **b** The structural features of FgMsn2 protein. FgMsn2 contains two ZnF_C2H2 domains (415–438 aa and 444–466 aa) and an NLS domain (522–531 aa). **c** Schematic drawing of ZnF_C2H2 domain of FgMsn2 in *F. graminearum* and other fungi

In *F. graminearum*, we identified a critical transcription factor FgMsn2, orthologous to Msn2 in *S*. *cerevisiae*. Phylogenetic analysis of Msn2 orthologs from various fungi, including *F. graminearum*, *Fusarium oxysporum*, *Trichoderma atroviride*, *B. bassiana*, *V. dahlia*, *M. oryzae*, *Neurospora crassa*, *B. cinerea*, *A. alternata*, *A. flavus*, *C. albicans* and *S. cerevisiae*, showed that FgMsn2 is most closely related to its ortholog in *F. oxysporum* (87.96% sequence identity), but evolutionarily distant from those in *S. cerevisiae* and *C. albicans* (9% to 12% identity) (Fig. 1a, Table 1).

**Table 1:** The amino acid identities of Msn2 orthologs and its zinc finger domain in different species

Species	Msn2 orthologs	Length (aa)	Full Length Sequence Identity (%)	Zinc Finger Domain Identity (%)
*F. graminearum*	FgMsn2	531	100	100
*F. oxysporum*	FoMsn2	535	87.96	100
*T. atroviride*	Seb1	532	64.56	100
*B. bassiana*	BbMsn2	488	53.21	100
*V. dahlia*	VdMsn2	577	56.44	90.38
*M. oryzae*	MoMsn2	548	44.79	90.38
*N. crassa*	Seb-1	575	36.25	88.46
*B. cinerea*	BcMsn2	601	41.82	90.38
*A. alternata*	AaSrr1	525	34.65	84.62
*A. flavus*	MsnA	608	34.05	94.23
*C. aibicans*	CaMsn4	757	10.44	61.54
*C. aibicans*	Mnl1	905	8.95	67.31
*S. cerevisiae*	Msn2	704	11.39	61.54
*S. cerevisiae*	Msn4	630	11.75	64.46

Multiple sequence alignments indicate that the FgMsn2 shares highly conserved typical features with other fungal Msn2 orthologs, including a nuclear localization signal (NLS) at residues 522–531 and two zinc finger domains (ZnF_C2H2) at residues 415–438 and 444–466 (Fig. 1b). The zinc finger region of FgMsn2 is significantly highly conserved, with over 60% sequence identity to the Msn2 ortholog in *S. cerevisiae* or *C. albicans*, despite their distant phylogenetic relationship (Fig. 1c, Table 1). However, the conservation of other regions is much lower (Table 1). These results suggest the conserved role of zinc finger domains in binding specific DNA motifs, while the non-conserved regions may have species-specific functions.

### FgMsn2 is important for vegetative growth and conidiation

**Fig. 2:**
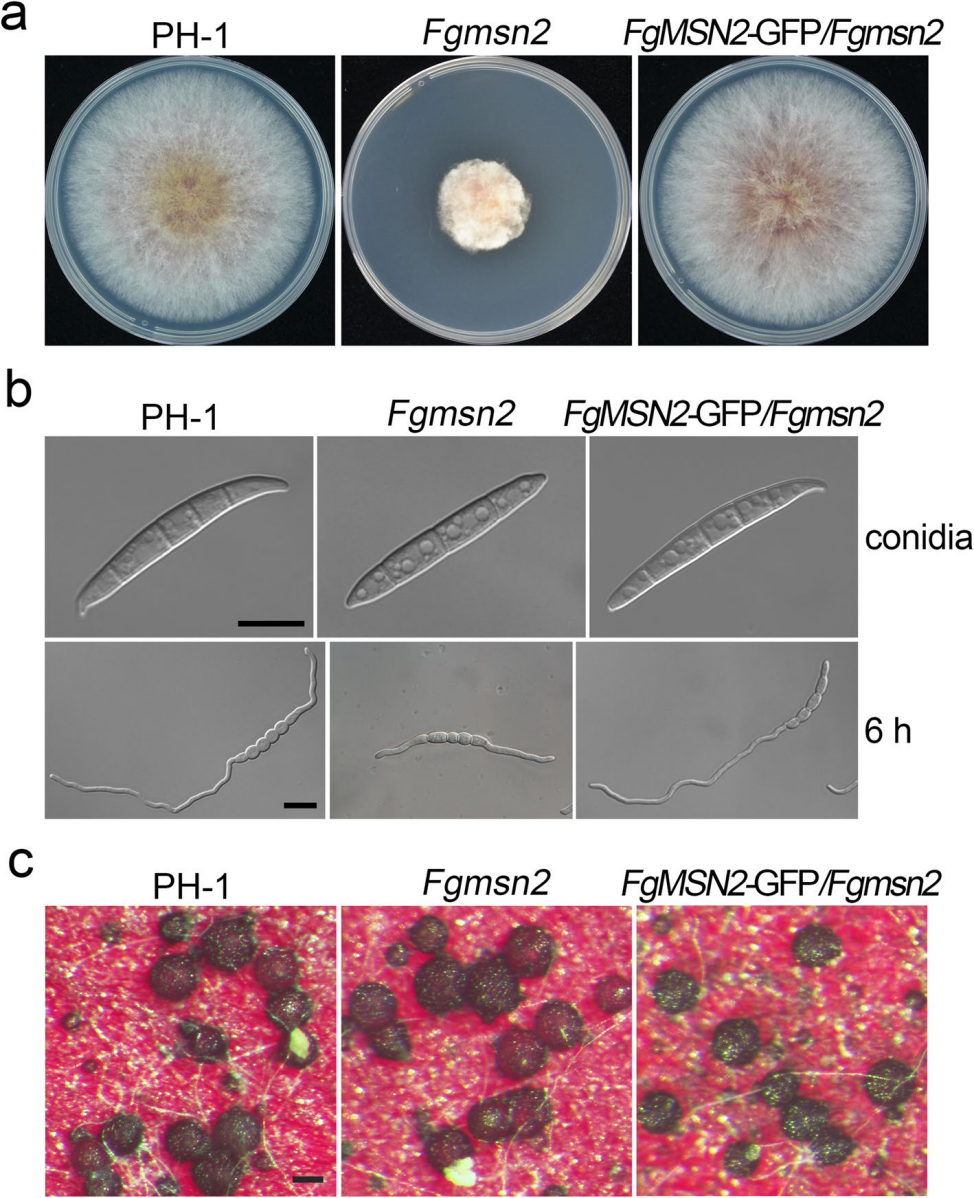
Defects of the *Fgmsn2* mutant in vegetative growth, conidiogenesis, and conidial germination **a** Three-day-old PDA cultures of the wild-type PH-1, *Fgmsn2* mutant and the complemented transformant *FgMSN2*-GFP/*Fgmsn2*. **b** Conidia and 6-h germlings of the same set of strains were observed by differential interference contrast (DIC) microscopy. Bar = 10 μm. **c** Perithecium formation of the indicated strains was examined on carrot cultures at 7 dpf. Bar = 100 μm

To determine the function of *FgMSN2*, we first generated the *Fgmsn2* mutants with a split-marker approach (Fig. S1a). The *Fgmsn2* deletion mutants with hygromycin resistance were confirmed by PCR analysis (Fig. S1b). When grown on PDA plates for 3 days, the *Fgmsn2* mutant formed compact and whitish colony with more fluffy aerial hyphae than PH-1. The growth rate of *Fgmsn2* mutant (4.96 mm/day) was reduced approximately by 61% compared to PH-1 (12.85 mm/day) (Fig. 2a, Table 2). These results indicate that FgMsn2 is important for vegetative growth and colony morphology in *F. graminearum*.

**Table 2:** Defects of the *Fgmsn2* mutant in vegetative growth, conidiation and plant infection

**Strains**	**Growth rate (mm/day)**^**A**^	**Conidiation (× 10**^**5**^**conidia/mL)**^**B**^	**Disease index**^**C**^
PH-1	12.85 ± 0.11^a^	109.14 ± 7.70^b^	9.11 ± 1.70^a^
*Fgmsn2*	4.96 ± 0.08^b^	130.43 ± 7.73^a^	1.28 ± 0.72^b^
*FgMSN2*-GFP/*Fgmsn2*	12.89 ± 0.21^a^	110.50 ± 8.48^b^	8.00 ± 1.73^a^

The means ± SE were calculated from the results of three independent experiments. Different letters denote significant differences determined by Fisher’s least significant difference (LSD) test at *P* < 0.05

^A^ The average growth rate was measured as the daily expansion of the colony radius after incubation for three days on PDA

^B^ Conidiation was quantified with 5-day-old cultures grown in carboxymethylcellulose (CMC) medium

^C^ Disease index was evaluated based on the number of symptomatic spikeletes 14 days after inoculation. Mean and standard errors were calculated from three independent experiments. At least 10 wheat heads were examined in each repeat

In addition, the *Fgmsn2* mutant showed a 19.5% increase in conidiation compared to PH-1 (Table 2). Additionally, we observed that most of the *Fgmsn2* conidia exhibited abnormal morphology. The conidia of the *Fgmsn2* mutant were straight, with fewer septa and shorter lengths compared to the wild-type conidia (Fig. 2b). Furthermore, the *Fgmsn2* mutant produced shorter germlings than PH-1 at 6 h post-inoculation (hpi) in YEPD at 25 °C (Fig. 2b). On carrot agar cultures, the *Fgmsn2* mutant formed abundant melanized perithecia as well as the PH-1 at 7 days post-fertilization (dpf) (Fig. 2c). For complementation assays, the *FgMSN2*-GFP fusion construct with its native promoter was generated and transformed into the *Fgmsn2* mutant. In the resulting *FgMSN2*-GFP/*Fgmsn2* transformant, the defects of *Fgmsn2* mutant in vegetative growth, and conidiation were fully restored (Fig. 2). These results indicate that *FgMSN2* is important for vegetative growth and conidiation.

### FgMsn2 is indispensable in plant infection and DON production

**Fig. 3:**
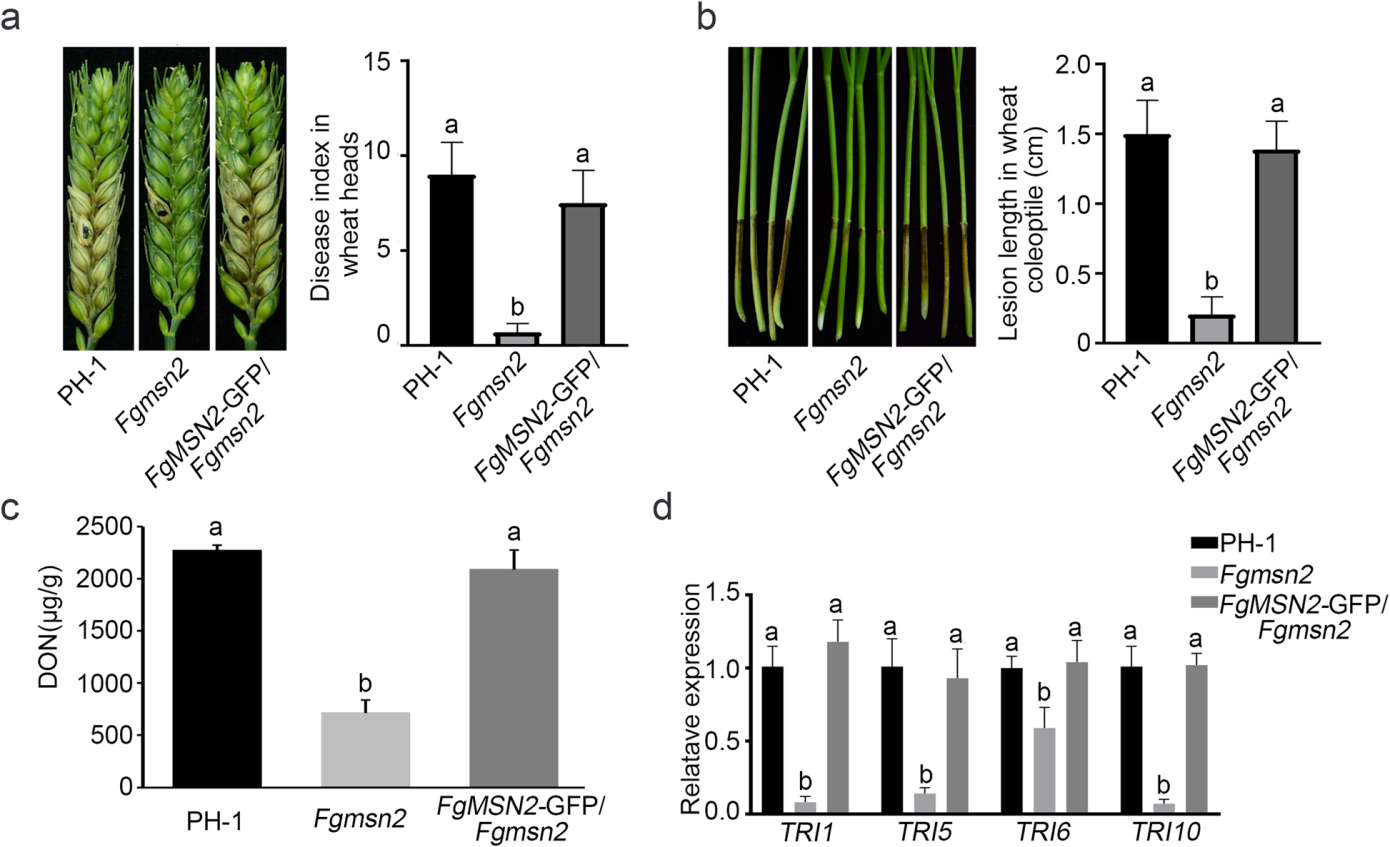
Defects of the *Fgmsn2* mutant in plant infection and DON synthesis **a**. Flowering wheat heads of cultivar Xiaoyan22 were drop-inoculated with conidia from wild-type PH-1, *Fgmsn2* mutant and *FgMSN2*-GFP/*Fgmsn2*. Spikelets with typical symptoms were photographed at 14 days post-inoculation (dpi). The inoculated spikelets were marked with a black dot. The bar chart illustrates the disease index of the indicated strains in infection assays performed on wheat heads at 14 dpi. **b**. Wheat coleoptiles inoculated with the same set of strains were photographed at 5 dpi. The bar chart shows the lesion length in wheat coleoptiles inoculated with the indicated strains. **c** DON production of the same set of strains were assayed in LTB cultures at 7 dpi. **d** The expression levels of *TRI1*, *TRI5*, *TRI6* and *TRI10* were determined by qRT-PCR using RNA isolated from indicated strains. The relative expression level of each gene in PH-1 was arbitrarily set to unity. The means and standard errors were calculated using data from three independent biological replicates. Letters on the bars indicate significantly differences determined by Fisher’s least significant difference (LSD) test at *P* < 0.05

We also assayed the defect of the *Fgmsn2* mutant in plant infection. The conidial suspensions of PH-1, *Fgmsn2* mutant and *FgMSN2*-GFP/*Fgmsn2* were drop-inoculated in the spikelets of the flowering wheat heads. At 14 days post-inoculation (dpi), both wild-type PH-1 and transformant *FgMSN2*-GFP/*Fgmsn2* caused typical scab symptoms in both the inoculated and the neighboring spikelets. However, the *Fgmsn2* mutant caused typical symptoms only restricted to the inoculated kernels, without spreading to adjacent spikelets (Fig. 3a). The *Fgmsn2* mutant also was reduced in virulence in infection assay with wheat coleoptiles. The *Fgmsn2* mutant developed necrosis lesions only at the inoculation sits at 5 dpi, whereas the PH-1 and complemented transformant *FgMSN2*-GFP/*Fgmsn2* caused longer lesions on the coleoptiles (Fig. 3b).

Since DON is an essential virulence factor in *F. graminearum*, we investigated the effect of *FgMSN2* deletion on DON production. In liquid trichothecene biosynthesis (LTB) cultures, the PH-1 strain produced DON at a concentration of 2,270.80 µg/g, while the *Fgmsn2* mutant produced only 721.35 µg/g (Fig. 3c), indicating an approximately 68% reduction in DON production. Furthermore, we analyzed the expression levels of *TRI* genes including *TRI1*, *TRI5*, *TRI6* and *TRI10*, and found that all these four genes were significantly down-regulated in the *Fgmsn2* mutant (Fig. 3d). In the complemented transformant *FgMSN2*-GFP/*Fgmsn2*, the defects in the pathogenicity and DON production were completely rescued (Fig. 3c). These results indicate that the *FgMSN2* plays an important role in pathogenicity and DON production in *F. graminearum*.

### FgMsn2 is mainly localized in nuclei in both conidia and hyphae

**Fig. 4:**
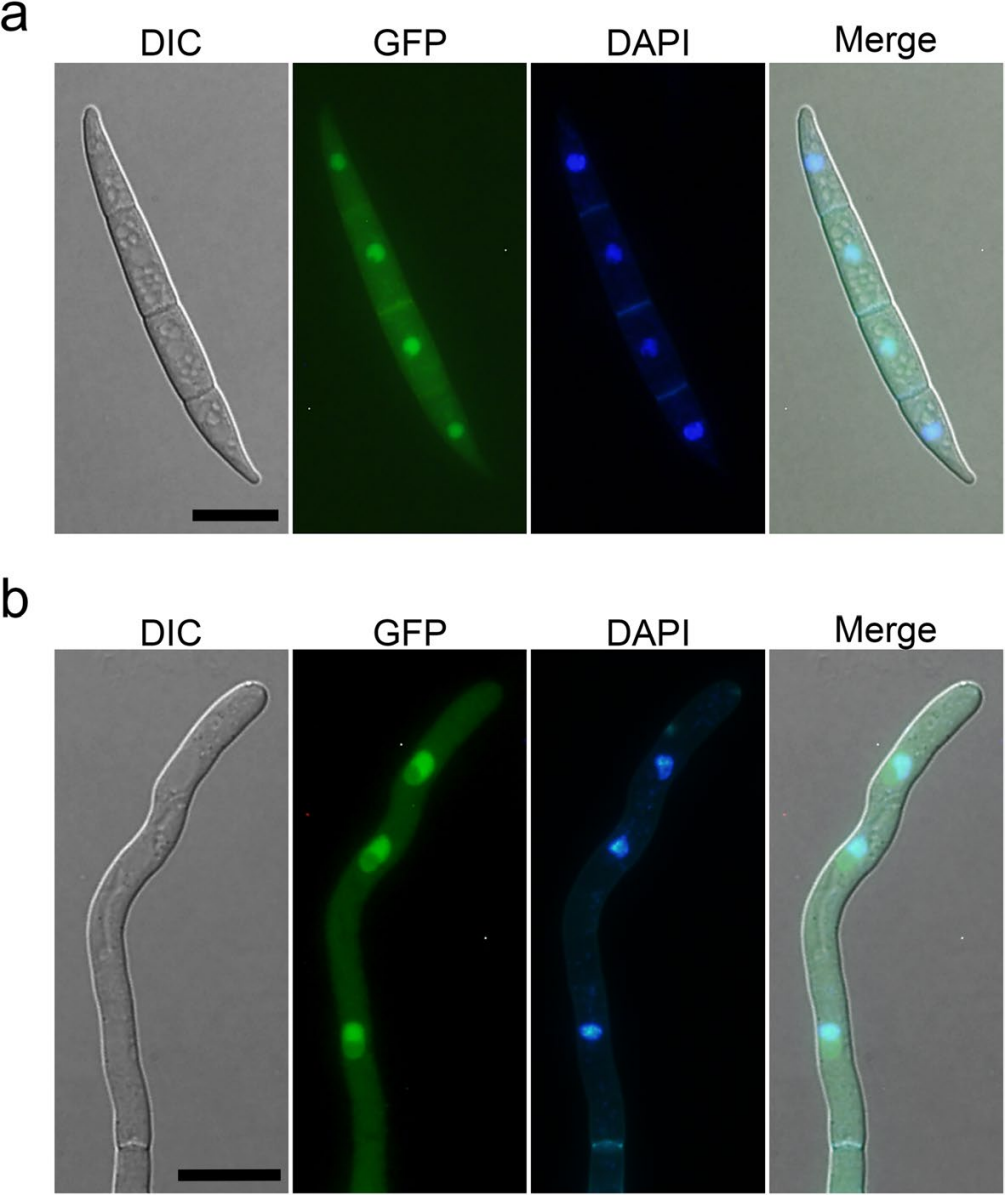
FgMsn2 is mainly localized into the nucleus in conidia and hyphae **a** Fresh conidia harvested from the complemented transformant *FgMSN2*-GFP/*Fgmsn2* were stained with 4,6-diamidino-2-phenylindole (DAPI) and examined by DIC and epifluorescence microscope. GFP signals were detected in both the nucleus and cytoplasm, but the fluorescence intensity was stronger in the nucleus. Bar = 10 μm. **b** The 12 h hyphae of *FgMSN2*-GFP/*Fgmsn2* transformant in YEPD medium were observed by DIC and epifluorescence microscope. Bar = 10 μm

The *FgMSN2*-GFP fusion construct is functional, since it successfully rescued the defects of the *Fgmsn2* mutant (Figs. 2 and 3). Thus, we examined the subcellular localization of FgMsn2 in the *FgMSN2*-GFP/*Fgmsn2* transformant. In conidia, strong GFP signals were predominantly observed in the nucleus, as confirmed by co-localization with dye 4’,6-diamidino-2-phenylindole (DAPI) staining, whereas weaker GFP signals were detected in the cytoplasm (Fig. 4a). Similar localization patterns were also observed in the hyphae, with GFP signals strongly concentrated in the nucleus and weaker signals present in the cytoplasm (Fig. 4b). These results indicate that FgMsn2 is localized mainly in the nucleus in *F. graminearum*.

### FgMsn2 plays a crucial role in response to environmental stresses

**Fig. 5:**
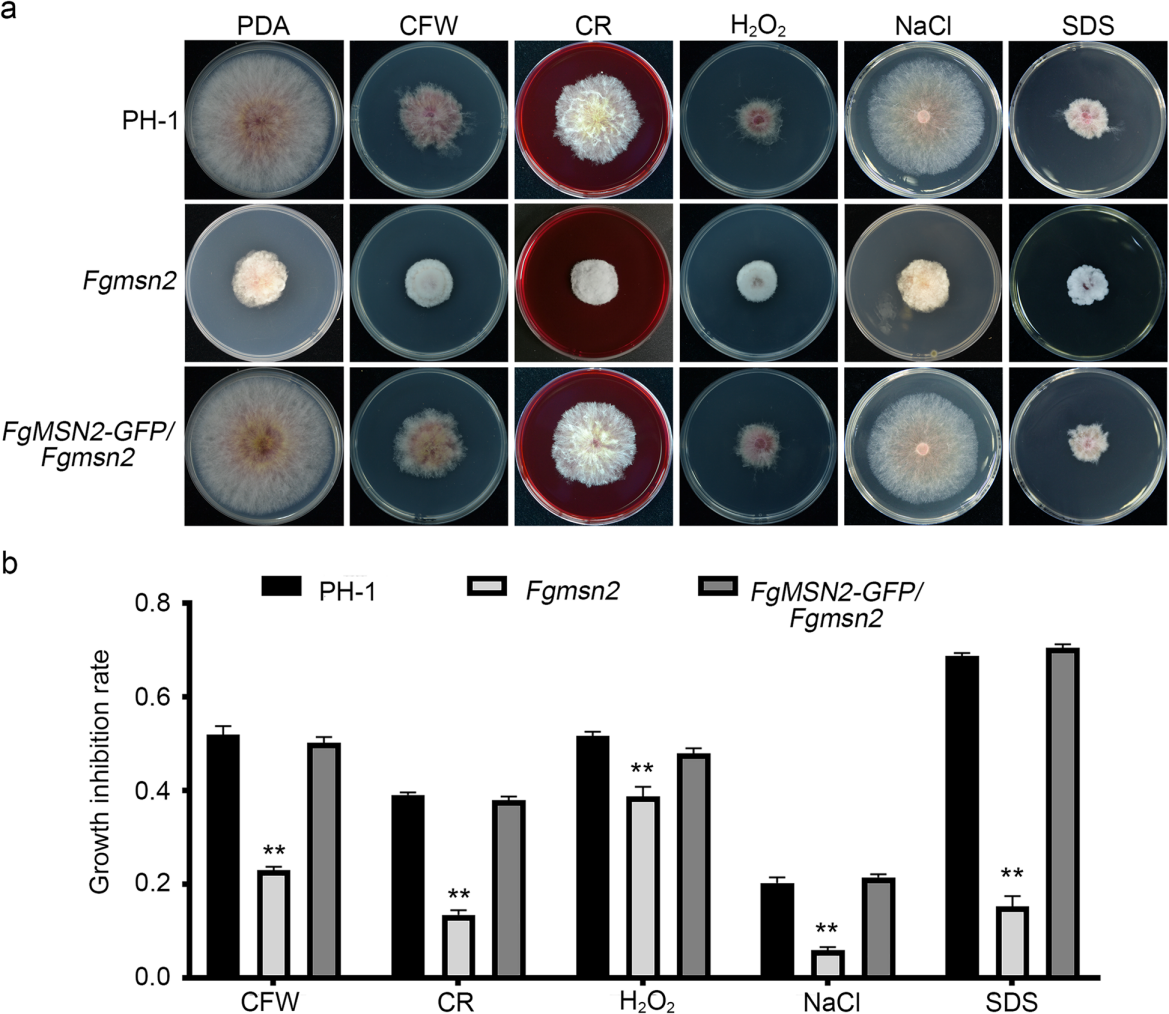
The sensitivity of *Fgmsn2* mutant in response to various stresses **a** The wild-type PH-1, *Fgmsn2* mutant and the complemented transformant *FgMSN2*-GFP/*Fgmsn2* were cultured on PDA plates with or without 300 µg/mL Calcofluor White (CFW), 300 µg/mL Congo Red (CR), 0.05% H_2_O_2_, 0.7 M NaCl and 0.05% SDS at 25 °C for 3 days. The *Fgmsn2* mutant displayed resistance to cell wall stresses (CFW and CR), oxidative stress (H_2_O_2_), osmotic stress (NaCl) and membrane stress (SDS). **b** The growth inhibition rates of the indicated strains under different stress conditions. Mean values ± SD were derived from three independent experiments. Statistically significant differences compared to the wild-type strain are indicated by asterisks (*P* < 0.01)

To determine whether FgMsn2 is involved in response to environment stresses, we grew the *Fgmsn2* mutant on PDA plates supplemented with different stress agents, including 300 μg/mL Calcofluor White (CFW), 300 μg/mL Congo Red (CR), 0.05% H_2_O_2_, 0.7 M NaCl and 0.05% SDS. After incubation at 25 °C for 3 days, the *Fgmsn2* mutant showed an increased resistance to CFW, CR, H_2_O_2_, NaCl and SDS in comparison to the wild-type PH-1 (Fig. 5). Moreover, these stress-related defects were rescued in the complemented transformant *FgMSN2*-GFP/*Fgmsn2* (Fig. 5). These results indicate that FgMsn2 plays an important role in response to various environmental stresses in *F. graminearium*.

### RNA-seq analysis reveals global changes in metabolism and stress responses in *Fgmsn2* mutant

**Fig. 6:**
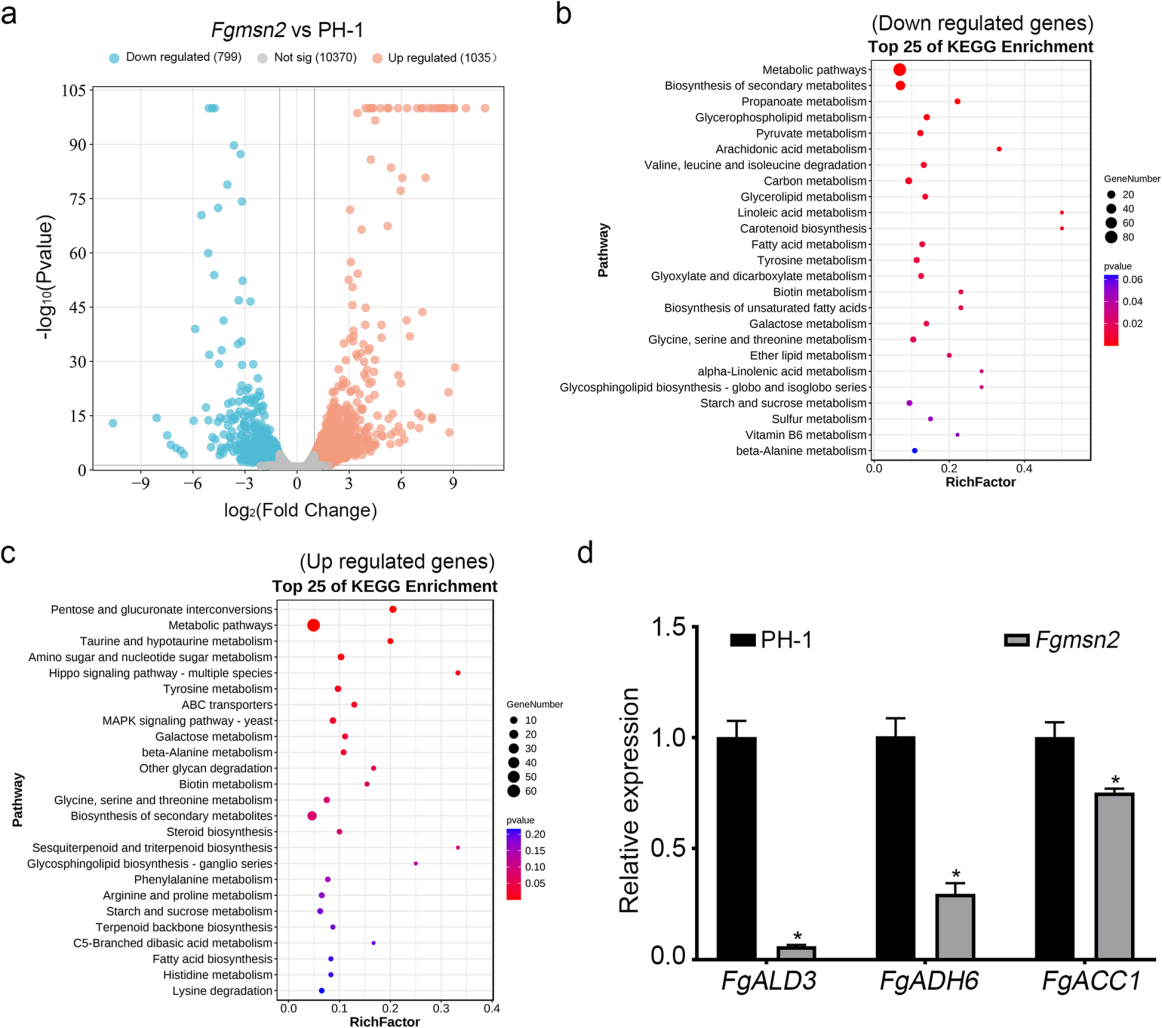
RNA-seq analysis of the wild-type PH-1 and *Fgmsn2* mutant **a** A volcano plot depicting the 799 and 1035 genes that were significantly upregulated (red dots) and downregulated (blue dots), respectively, in the *Fgmsn2* mutant in comparison with the wild-type PH-1. **b** and **c** represent the top 20 KEGG enrichment pathways of significantly down-regulated and up-regulated genes in *Fgmsn2* mutant, respectively. **d** The expression levels of *FgALD3*, *FgADH6* and *FgACC1* were determined by qRT-PCR using RNA isolated from PH-1 and *Fgmsn2* mutant. The relative expression level of each gene in PH-1 was arbitrarily set to unity. Statistically significant differences compared to the wild-type strain are indicated by asterisks (*P* < 0.05)

To further determine the function of FgMsn2, RNA-seq analysis was performed to compare global gene expression patterns between the *Fgmsn2* mutant and wild-type PH-1. A total of 1,834 differentially expressed genes were identified, with 799 downregulated and 1,035 upregulated in the *Fgmsn2* mutant (Fig. 6a). Specifically, KEGG pathway enrichment analysis showed that the downregulated genes were primarily associated with energy and lipid metabolism. The key energy metabolism pathways, including propanoate metabolism, pyruvate metabolism, carbon metabolism, glyoxylate and dicarboxylate metabolism, and starch and sucrose metabolism, were significantly enriched (Fig. 6b). Similarly, the lipid metabolism pathways, such as glycerophospholipid metabolism, arachidonic acid metabolism, linoleic acid metabolism, fatty acid metabolism, biosynthesis of unsaturated fatty acids and ether lipid metabolism, were also significantly affected (Fig. 6b). These results indicate that *FgMSN2* plays a critical role in maintaining energy and lipid metabolic processes. Moreover, the upregulated genes in the *Fgmsn2* mutant are primarily involved in cell wall structural synthesis, stress responses, and secondary metabolism (Fig. 6c). These include pathways such as amino sugar and nucleotide sugar metabolism, glycan degradation, β-alanine metabolism, MAPK signaling, and ABC transporters. In addition, upregulated expression is observed in lysine degradation and phenylalanine metabolism. Furthermore, genes related to steroid biosynthesis, glycosphingolipid biosynthesis, and fatty acid biosynthesis are also upregulated.

To verify the reliability of the RNA-seq data, three key genes associated with pyruvate metabolism were selected for analysis by qRT-PCR, including *FgALD3* (aldehyde dehydrogenase), *FgADH6* (alcohol dehydrogenase), and *FgACC1* (acetyl-CoA carboxylase). Consistent with the RNA-seq results (Table S2), all three genes showed significant downregulation, with *FgALD3* and *FgADH6* decreasing over twofold (Fig. 6d). Therefore, *FgMSN2* serves as a critical regulator of metabolism and stress responses in *F. graminearum*.

### FgMsn2 is required for the maintenance of mitochondrial morphology

**Fig. 7:**
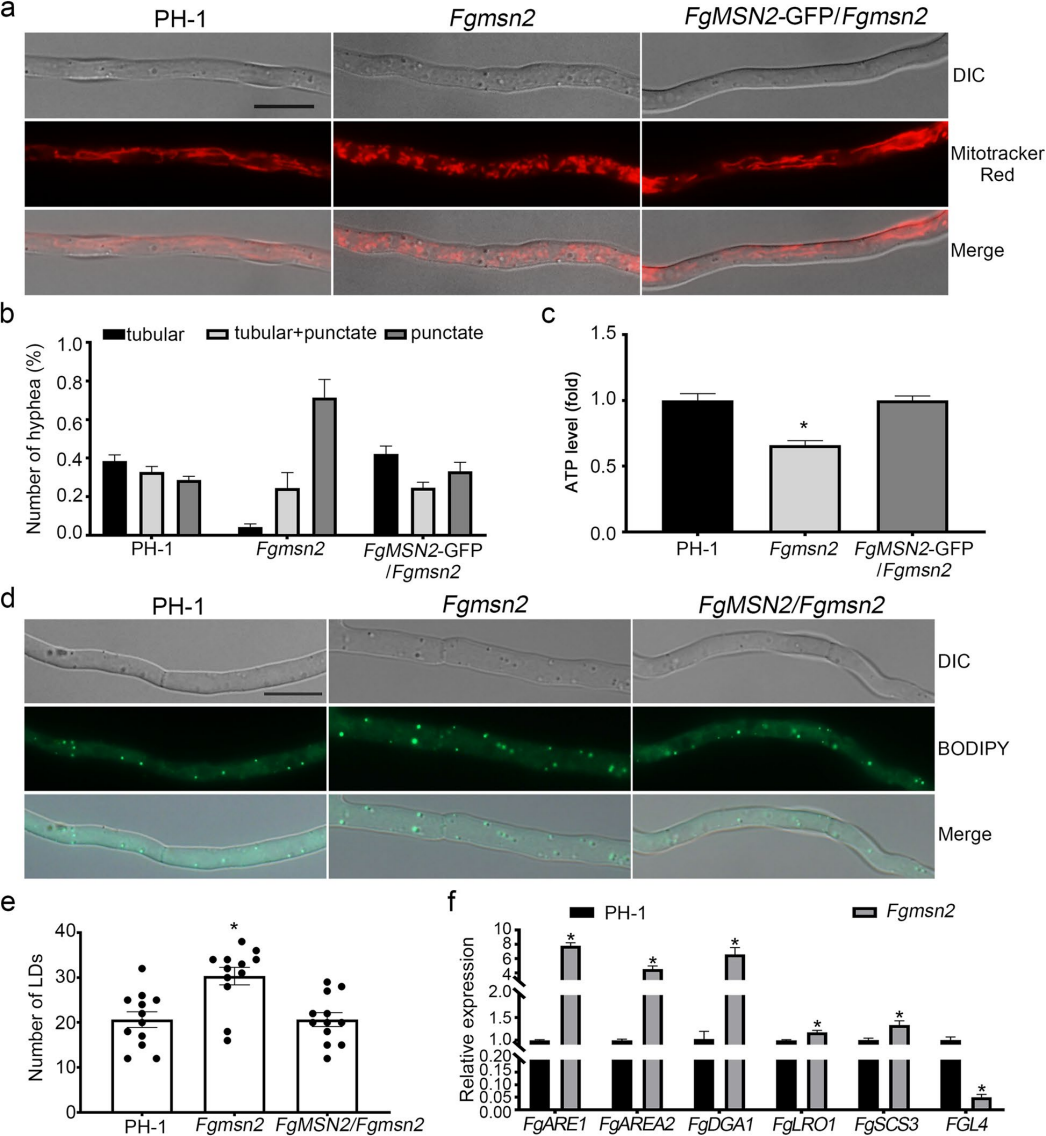
FgMsn2 plays a critical role in mitochondrial morphology and lipid droplet accumulation in F. graminearum **a** Mitochondria in hyphae of the wild-type PH-1, the Fgmsn2 mutant and the complemented transformant FgMSN2-GFP/Fgmsn2 were stained with MitoTracker Red and visualized using an Olympus BX53 fluorescence microscope. Bar = 10 μm. **b** Statistical analysis of the mitochondrial morphology in the indicated strains. The mean values ± SD were calculated from three independent experiments. **c** Relative ATP level of PH-1 and Fgmsn2 mutant. **d** The morphology and number of lipid droplets were examined and statistically analyzed (**e**) in the same set of strains, stained with BODIPY. Bar = 100 μm. Error bars represent standard deviations (SDs) from three biological replicates, and asterisks indicate significant differences determined by one-way ANOVA (*P* < 0.05)

To investigate whether FgMsn2 is involved in energy metabolism, we examined the mitochondrial morphology by staining the vegetative hyphae of PH-1, *Fgmsn2* mutant and complemented transformant *FgMSN2*-GFP/*Fgmsn2* with MitoTracker Red. Under microscopic examination, both tubular and punctate mitochondrial morphologies were observed in the hyphae of the wild-type PH-1, *Fgmsn2* mutant, and complemented transformant *FgMSN2-*GFP*/Fgmsn2*. However, a detailed analysis revealed a significant difference: while 40% mitochondria were tubular in the hyphae of PH-1, only 5% mitochondria exhibited a tubular morphology in the *Fgmsn2* mutant (Fig. 7a, b). Furthermore, we measured the ATP content in the hyphae of the *Fgmsn2* mutant and found that the ATP level in the *Fgmsn2* mutant was significantly lower than those in PH-1 and *FgMSN2-*GFP*/Fgmsn2* (Fig. 7c), suggesting impaired mitochondrial function. These results indicate that FgMsn2 is critical for maintaining mitochondrial morphology and ATP production in *F. graminearum.*

### FgMsn2 is involved in lipid droplet accumulation

Our RNA-seq data showed that the deletion of the *FgMSN2* gene significantly changed the transcriptional levels of numerous lipid metabolic genes (Fig. 6). Additionally, the *FgMSN2* deletion disrupted mitochondrial morphology (Fig. 7), which is critical for β-oxidation of fatty acid. Thus, we hypothesize that FgMsn2 play a critical role in regulating lipid homeostasis, particularly in lipid droplet dynamics. To test this hypothesis, we examined lipid droplet accumulation in the *Fgmsn2* mutant. When stained with BODIPY, we observed that the morphology of lipid droplets remained unchanged, but their number was significantly increased in the *Fgmsn2* mutant (Fig. 7d, e). As the previously generated complemented transformant *FgMSN2-*GFP*/Fgmsn2* contained a GFP tag that could interfere with BODIPY detection, we generated a new complemented transformant, *FgMSN2/Fgmsn2*, without the GFP tag. Microscopic analysis confirmed that the lipid droplet number in *FgMSN2/Fgmsn2* transformant was restored to the wild-type level (Fig. 7d, e). Consistent with the this, RNA-seq data (Table S2) showed that significant upregulation of lipid droplet biogenesis genes (*FgARE1*, *FgARE2*, and *FgDGA1*), along with marked downregulation of the triacylglycerol lipase gene *FGL4* responsible for hydrolyzing stored triacylglycerols within lipid droplets. These transcriptional changes were validated by qRT-PCR (Fig. 7f). These results indicate that FgMsn2 is important for lipid droplet accumulation in *F. graminearum* by regulating both lipid droplet biosynthesis and degradation pathways.

## Discussion

Fungi have developed sophisticated regulatory systems to adapt to various environmental stresses, in which transcription factors play a critical role by regulating gene expression. Among these, Msn2/Msn4, a zinc finger DNA-binding protein, serves as a key transcriptional activator for multi-stress responses by binding to the STRE sequence “CCCCT” in the promoter regions of downstream genes (Martinez‐Pastor et al., 1996).

In this study, we identified a putative Msn2 ortholog, FgMsn2, in *F. graminearum*. Deletion of *FgMSN2* led to a significant reduction in vegetative growth but a substantial increase in conidiation. This result is consistent with what has been reported in some fungi such as *A. flavus* and *A. alternata* (Chang et al. 2011; Lu et al. 2022). However, in *Alternaria brassicicola*, the Msn2 ortholog Abvf19 is dispensable for vegetative growth (Srivastava et al. 2012). Interesting, in *M. orzyae*, *B. cinerea* and *U*. *virens*, the deletion of *MSN2* orthologs results in a reduction in both vegetative growth and conidiation (Zhang et al. 2014; Xu et al. 2021; Lu et al. 2023), suggesting the functional divergence of Msn2 orthologs. Furthermore, recent studies have revealed that the transcription factor FgCon7 in *F. graminearum* directly binds to the promoter region of *FgMSN2*, thereby regulating the growth and conidiation (Chen et al. 2024). Therefore, Msn2 plays distinct roles in fungal growth and conidiation depending on the fungal species.

In *S. cerevisiae*, Msn2 and Msn4 regulate the transcription of stress-related genes, enabling cells to respond to various environmental stresses. Similarly, in *B*. *bassiana* and *Metarhizium robertsii*, Msn2 regulates responses to various environmental stresses (Liu et al. 2013). However, in *C*. *albicans* and *V*. *dahliae*, the Msn2 does not appear to participate in any stress responses (Nicholls et al. 2004; Tian et al. 2017), suggesting that they may rely on alternative signaling pathways or transcription factors for stress adaptation. Interestingly, in *A*. *alternata*, deletion of the Msn2 ortholog (*AaSRR1*) enhances tolerance to multiple oxidative stresses as well as cell wall (CFW, Congo red) and membrane stresses (SDS, Triton X-100) (Lu et al. 2022). Similarly, in our study, the *Fgmsn2* deletion mutant also displayed increased tolerance to oxidative, osmotic, cell wall, and membrane stresses, further highlighting the diverse roles of Msn2 proteins in stress responses across fungal species. Both *F. graminearum* and *A. alternata* are necrotrophic plant pathogens with similar infection strategies, relying on toxin production for infection, which may account for the similar stress response behaviors in their *msn2* deletion mutants. These enhanced stress tolerances of the *Fgmsn2* mutant may result from its transcriptional reprogramming toward stress adaption. The upregulated pathways include those for cell wall remodeling (amino sugar and nucleotide sugar metabolism, glycan degradation), antioxidant defense (β-alanine metabolism), osmotic response (MAPK signaling, ABC transporters), and membrane reinforcement (steroid and glycosphingolipid biosynthesis), collectively enhancing resistance to cell wall, oxidative, osmotic, and membrane stresses.

Structural analysis further revealed that although Msn2 proteins exhibit low conservation across their full length, the C2H2 zinc-finger domain is highly conserved, likely to ensure efficient binding to the STRE motif. Notably, from yeast to filamentous fungi including *S. cerevisiae*, *Y. lipolytica*, *T. atroviride*, and *N. crassa*, Msn2 orthologs consistently bind to the STRE motif (Martinez‐Pastor et al., 1996; Hurtado and Rachubinski 1999; Peterbauer et al. 2002; Freitas et al. 2016; Chen et al. 2021), indicating this binding ability is highly conserved. Furthermore, the conserved C2H2 zinc-finger domain and NLS in FgMsn2 strongly suggest its role as a transcriptional regulator in *F. graminearum*. Consistently, we demonstrated that the FgMsn2 predominantly localizes in the nucleus under normal conditions. In contrast, in *S. cerevisiae* and *A. alternata*, Msn2 is localized in the cytoplasm but translocates to the nucleus under stress conditions (Görner et al. 1998; Lu et al. 2022). This difference in nuclear-cytoplasmic distribution pattern of Msn2 suggest different regulatory mechanisms across fungal species. In *S. cerevisiae*, the nuclear translocation of Msn2 is negatively regulated by the PKA signaling pathway (Görner et al. 1998). However, such regulation mechanism does not appear to be conserved in filamentous fungi. For instance, the RXXS/T motifs characteristic of PKA phosphorylation are absent in Seb1 of *T. atroviride* (Peterbauer et al. 2002). In contrast, in *M. oryzae*, the cAMP-PKA signaling pathway positively regulates the nuclear localization of MoMsn2 (Zhang et al. 2023).

Beyond regulating stress responses, Msn2 orthologs play key roles in pathogenicity, and secondary metabolism. In *F. graminearum*, deletion of *MSN2* caused a significant reduction in pathogenicity, highlighting its critical role in virulence. Similarly, *MSN2* orthologs are indispensable for pathogenicity in various plant and animal pathogenic fungi, including *M. oryzae*, *B. cinerea*, *A. alternata*, *B. bassiana* and *Arthrobotrys oligospora* (Liu et al. 2013, 2024; Zhang et al. 2014; Lu et al. 2022, 2023). However, in *C*. *glabrata*, the *CgMSN2* and *CgMSN4* have no effect on virulence during *Drosophila* infection, possibly due to the absence of extreme stress conditions in the fly hemocoel (Roetzer et al. 2008). Therefore, although Msn2/4 orthologs are generally crucial for fungal pathogenicity, their significance may vary depending on the host-specific stress conditions.

In our study, deletion of *FgMSN2* also significantly reduced DON production and downregulated the expression of *TRI* genes (*TRI1*, *TRI5, TRI6 and TRI10*), suggesting a direct role in trichothecene biosynthesis. This reduction in DON synthesis likely contributes to the reduced pathogenicity observed in the *Fgmsn2* mutant. Similarly, in *U*. *virens*, deletion of *UvMSN2* also leads to a decrease in the expression of several ustilaginoidin biosynthetic-related genes (Xu et al. 2021). In contrast, the loss of *MSNA* gene in *A. flavus* increases the production of aflatoxins and kojic acid, indicating its function as a negative regulator. Likewise, in *M*. *rileyi*, the MrMsn2 negatively regulates the pigment biosynthesis, which plays a crucial role in protecting the fungus against stress conditions (Song et al. 2018). Therefore, the Msn2 may have a broad and different function in secondary metabolite biosynthesis across different fungal species.

Our RNA-seq analysis reveals that FgMsn2 regulates energy and lipid metabolisms and stress adaptation. Consistently, we demonstrated that FgMsn2 regulates mitochondrial morphology and lipid metabolism. The *Fgmsn2* mutant exhibited disrupted mitochondrial structure and reduced ATP levels, linking FgMsn2 to mitochondrial function. Similar results have been reported in *M. oryzae* and* B*. *cinerea* (Xiao et al. 2021; Lu et al. 2023), suggesting a conserved role for Msn2 orthologs in mitochondrial function. In plant pathogens, the mitochondria play an essential role in plant infection (Zhong et al. 2016; Kang et al. 2022). Therefore, the mitochondrial dysfunction likely contributes significantly to the reduced virulence of the *Fgmsn2* mutant. Furthermore, we demonstrated that FgMsn2 regulates lipid metabolism, a process critical for energy storage, membrane biogenesis, and the production of signaling molecules. Similar regulatory roles of Msn2 in lipid metabolism have also been documented in *S. cerevisiae* and *M. oryzae* (Rajvanshi et al. 2017; Zhang et al. 2023). Since proper lipid regulation is vital for virulence in fungal pathogen (Wang et al. 2007; Patkar et al. 2012), the abnormal lipid metabolism may explain the reduced virulence in *Fgmsn2* mutants. Moreover, the roles of FgMsn2 in mitochondrial function and lipid metabolism may be closely intertwined with its functions in stress adaptation. Mitochondrial dysfunction can exacerbate ROS accumulation, thereby activating stress responses (Zadrąg-Tęcza et al. 2018), while disrupt lipid metabolism alerts the composition of the cell membrane (Peng and Chen 2024). These interconnected functions suggest that FgMsn2 integrates metabolic regulation with stress responses and pathogenicity, enhancing fungal adaptability.

Taken together, FgMsn2 is a central regulatory hub in *F. graminearum*, governing fungal development, stress responses, metabolic regulation and pathogenicity. Its role in maintaining mitochondrial function and lipid homeostasis highlights its critical contribution to both fungal development and virulence.

## Conclusion

In summary, our study demonstrates that FgMsn2, a transcription factor in *F. graminearum*, regulates fungal development, pathogenicity and stress adaptation. While its C2H2 zinc-finger domain is evolutionarily conserved across fungi, divergence in other structural regions indicates species-specific functional adaptations. Deletion of *FgMSN2* severely reduces vegetative growth, conidiation, DON biosynthesis, and pathogenicity, yet unexpectedly enhances tolerance to multiple environmental stresses including oxidative, osmotic, and membrane stresses. RNA-seq analyses link FgMsn2 to energy/lipid metabolism and stress responses, supported by disrupted mitochondrial integrity, reduced ATP, and increased lipid droplet accumulation in *Fgmsn2* mutant. These findings not only elucidate the critical roles of FgMsn2 in pathogenicity and stress adaptation but also highlight its potential as a promising target for novel antifungal strategies.

## Materials and methods

### Bioinformatics analyses

The protein sequence of Msn2 in *S. cerevisiae* was obtained from the SGD database (https://www.yeastgenome.org/). Subsequently, this sequence was used to perform a BLASTP search in the NCBI database (https://www.ncbi.nlm.nih.gov/) to identify the FgMsn2 in *F. graminearum*. Furthermore, orthologs of Msn2 from other representative fungi were identified through Ensembl Fungi (http://fungi.ensembl.org/index.html) by using the BLASTP algorithm. To analyze these orthologs, multiple sequence alignment of FgMsn2 orthologs was generated using MAFFT and visualized with ESPript 3.0 (https://espript.ibcp.fr/ESPript/ESPript/index.php). In the visual representation, identical amino acid residues were displayed as white text on a red background, while similar residues were shown as red text on a white background. The protein domains of FgMsn2 were predicted using the Pfam database (http://pfam.xfam.org/) and the nuclear localization signal (NLS) was predicted using the NLStradamus online tool (http://www.moseslab.csb.utoronto.ca/NLStradamus/).

### Fungal strains and culture conditions

All strains were routinely cultured on potato dextrose agar (PDA) plates at 25 °C. Colony diameters were measured on PDA plate to determine vegetative growth rates. Conidiation was assessed with 5-day-old CMC cultures, while sexual reproduction was examined on carrot agar plates, as described previously (Ding et al. 2009; Ni et al. 2024). Protoplast preparation and fungal transformations were conducted as described (Hou et al. 2002). Transformants were selected on TB3 medium supplemented with 300 µg/mL hygromycin B (MDbio, China) or 400 µg/mL G418 (Sigma-Aldrich, USA), respectively (Liang et al. 2021).

### Generation of *Fgmsn2* mutants

The *Fgmsn2* mutants were generated using the split-marker approach, as described previously (Wang et al. 2012). A 0.63-kb upstream and a 0.66-kb downstream fragments were amplified with primer pairs MSN2-1F/2R and MSN2-3F/4R, respectively. Two fragments of the *hph* gene (H1 and H2) were amplified using primer pairs HYG-F/HY-R and YG-F/HT-R, respectively. The upstream and downstream fragments of *FgMSN2* were fused with the H1 and H2 fragments of *hph* via overlapping PCR and transformed into PH-1. Transformants were selected on TB3 medium containing 300 µg/mL hygromycin B and screened by PCR for deletion of the *FgMSN2* gene.

### Generation of *FgMSN2*-GFP and *FgMSN2*-C constructs

For the *FgMSN2*-GFP and *FgMSN2*-C constructs, the *FgMSN2* gene with its native promoter, was amplified using primers MSN2-GF and MSN2-GR, MSN2-F and MSN2-R, respectively (Table S1). The PCR product was cloned into the *Kpn* I/*Hind* III double-digested pKNTG vector by using the ClonExpress® II One Step Cloning Kit (Vazyme, China). These constructs were verified by sequencing analyses.

### Plant infection and DON production assays

Conidia were harvested from 5-day-old CMC cultures and suspended in sterile water to a final concentration of 2 × 10^5^ spores/mL for plant infection assays. Flowering wheat heads (Xiaoyan22) were inoculated with 10 µL of the conidial suspension at the fifth spikelet from the base of the spike, following previously described methods (Wang et al. 2018). Disease symptoms were assessed at 14 dpi by counting the number of symptomatic spikelets per wheat head (Zhou et al. 2010). For DON production assays, conidia were resuspended to a final concentration of 10^4^ conidia/mL in LTB medium. After incubation for 7 days, DON production was measured by GCMS-QP2010 system with AOC-20i autoinjector (Shimadzu Co., Japan) (Huang et al. 2024). For each strain, mean and standard deviation were calculated from data obtained from three biological replicates.

### Assays for defects in response to abiotic stresses

The final concentration of 300 μg/mL CFW, 300 μg/mL CR, 0.05% H_2_O_2_, 0.7 M NaCl and 0.05% SDS was added to PDA to assay for colony growth at 25 °C as described (Ren et al. 2019). Colony morphology was examined and photographed after 3 days of incubation. The inhibition rate is calculated as the percentage reduction in colony radius on stressed PDA compared to that on regular PDA.

### RNA-seq analysis

Mycelia of the wild-type strain PH-1 and the *Fgmsn2* mutant were collected after 3 days of incubation on PDA plates. Total RNA was extracted using the Oligotex mRNA Mini Kit (Qiagen, Germany), with three biological replicates for each strain. The libraries were constructed and sequenced on an Illumina HiSeq-2500 platform (2 × 150 bp paired-end reads) at the Novogene Bioinformatics Institute (Beijing, China). The RNA-seq reads were aligned to the PH-1 reference genome using Hisat2 (Kim et al. 2015), and featureCounts was used to quantify transcript abundance (Liao et al. 2014). Differentially expressed genes (DEGs) were identified with edgeRun (Dimont et al. 2015), with thresholds of > twofold upregulation or < 0.5-fold downregulation (*P* < 0.05). Kyoto Encyclopedia of Genes and Genomes (KEGG) enrichment analysis was performed using the OmicShare tool (https://www.omicshare.com/tools/home/report/koenrich.html).

### Quantitative reverse transcription-polymerase chain reaction (qRT-PCR) analysis

Conidia harvested from five-day-old CMC cultures were transferred to LTB medium, and after three days of incubation, mycelia of the wild-type PH-1, *Fgmsn2* mutants and complemented transformant *FgMSN2*-GFP/*Fgmsn2* were collected for RNA isolation by using Eastep Super Total RNA Extraction Kit (Promega, Madison, WI, USA). Reverse transcription was performed using M-MLV GIII Reverse Transcriptase (Yugong Biotech, Jiangsu, China). The qRT-PCR test used the Taq-HS Probe qPCR Premix (Universal) (Yugong Biotech, Jiangsu, China). Primer pairs for amplifying *TRI1*, *TRI5*, *TRI6* and *TRI10* were TRI1-F/TRI1-R, TRI5-F/TRI5-R, TRI6-F/TRI6-R and TRI10-F/TRI10-R, respectively.

Total RNA was extracted from 12-h germinated hyphae of both wild-type PH-1 and *Fgmsn2* mutant strains. Following reverse transcription, the expression levels of *FgALD3*, *FgADH6*, *FgACC1*, *FgARE1*, *FgARE2*, *FgDGA1*, *FgLRO1*, *FgSCS3* and *FGL4* genes were analyzed by qRT-PCR using gene-specific primer pairs (FgALD3-F/R, FgADH6-F/R, FgACC1-F/R, FgARE1-F/R, FgARE2-F/R, FgDGA1-F/R, FgLRO1-F/R, FgSCS3-F/R and FGL4-F/R). The *ACTIN* gene (FGSG_07335) was used as the internal control. Relative expression levels of individual genes or transcripts were calculated using the 2^−ΔΔCt^ method (Livak and Schmittgen 2001). Data from three independent biological replicates were used to calculate the mean and standard deviation. All the primers used were listed in Table S1.

### Microscopic observation

For subcellular localization, the complemented transformant expressing *FgMSN2*-GFP was used. Hyphae and conidia were stained with the nucleus-specific dye 4’,6-diamidino-2-phenylindole (DAPI) at a concentration of 10 μg/mL for 5 min at room temperature in darkness as previously described (Li et al. 2015), then examined using an Olympus BX53 fluorescence microscope. For mitochondrial staining, hyphae and conidia of the indicated strains were stained by the mitochondria-specific marker Mito Tracker Red CMXRos (Beyotime, China) at concentration of 200 nM at room temperature for 1–2 min in darkness, and observed under an Olympus BX53 fluorescence microscope. For lipid droplet staining, hyphae cultured in liquid YEPD for 12 h were stained by BODIPY 493/530 (Beyotime, Shanghai, China) at final concentration of 1 μg/mL for 3 min in darkness.

## Supplementary Information

Supplementary Material 1. Fig. S1. The *FgMSN2* gene replacement construct and mutants (a). The *FgMSN2* locus and gene replacement construct. The *FgMSN2* and* hph* genes were indicated with empty and red arrows, respectively. Two pairs of primers MSN2-1F + MSN2-2R, and MSN2-3F + MSN2-4R were used to amplify the flanking sequences. (b). PCR analysis confirming the deletion of the *MSN2* gene. The mutants *Fgmsn2*-1, *Fgmsn2*-5, and *Fgmsn2*-8 were validated using four pairs of primers: MSN2-5F + MSN2-6R (L1, 852 bp), MSN2-7F + HT856R (L2, 2051 bp), and H855F + MSN2-8R (L3, 1293 bp), H850 + H852 (L4, 750 bp).

Supplementary Material 2. Table S1 PCR primers used in this study.

Supplementary Material 3. Table S2 Genes differently expressed in *Fgmsn2 *mutant.

## Data Availability

All data generated or analyzed during this study are included in the published article and its supplementary information files.

## Abbreviations

CFW: Calcoflour White
CR: Congo Red
DAPI: 4’,6-Diamidino-2-phenylindole
DON: Deoxynivalenol
dpf: Days post-fertilization
dpi: Days post-inoculation
hpi: Hours post-inoculation
LTB: Liquid trichothecene biosynthesis
NLS: Nuclear localization signal
PDA: Potato dextrose agar
STREs: Stress response elements
ZEN: Zearalenone
ZnF_C2H2: Zinc finger domains
