# Supplementary figures and images for: FgMsn2, a zinc finger transcription factor, regulates stress responses, pathogenicity and metabolism in wheat scab fungus Fusarium graminearum
Source: Stress Biol. 2025 Sep 2;5(1):54. doi: 10.1007/s44154-025-00249-2 (PMC12401790; doi:10.1007/s44154-025-00249-2)

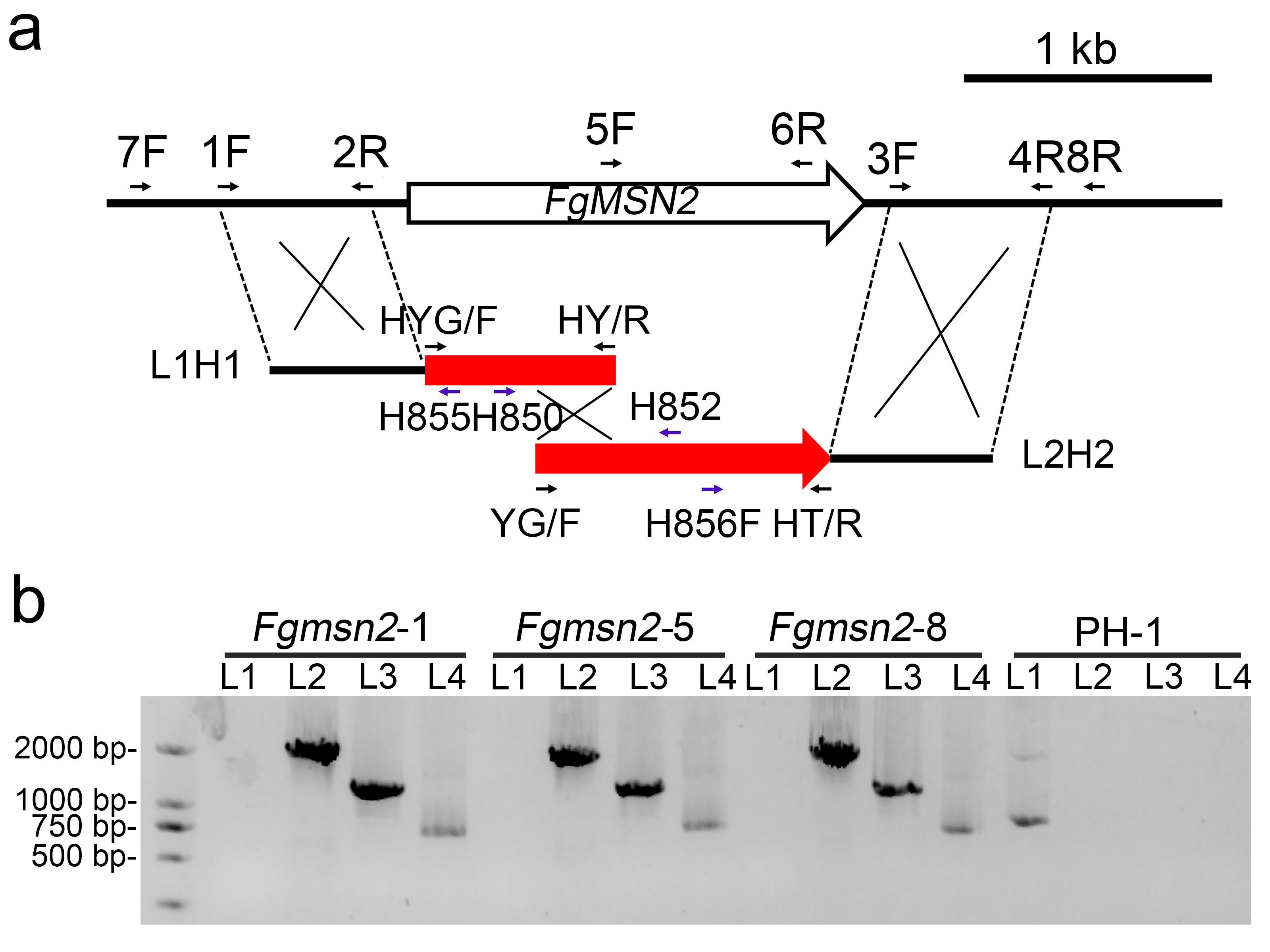

Supplement: Supplementary file 1 — Supplementary Material 1. Fig. S1. The FgMSN2 gene replacement construct and mutants (a). The FgMSN2 locus and gene replacement construct. The FgMSN2 and hph genes were indicated with empty and red arrows, respectively. Two pairs of primers MSN2-1F + MSN2-2R, and MSN2-3F + MSN2-4R were used to amplify the flanking sequences. (b). PCR analysis confirming the deletion of the MSN2 gene. The mutants Fgmsn2-1, Fgmsn2-5, and Fgmsn2-8 were validated using four pairs of primers: MSN2-5F + MSN2-6R (L1, 852 bp), MSN2-7F + HT856R (L2, 2051 bp), and H855F + MSN2-8R (L3, 1293 bp), H850 + H852 (L4, 750 bp). [file 44154_2025_249_MOESM1_ESM.tif]
